# Supplementary material for: The Systems Biology Research Tool: evolvable open-source software
Source: BMC Syst Biol. 2008 Jun 29;2:55. doi: 10.1186/1752-0509-2-55 (PMC2446383; doi:10.1186/1752-0509-2-55)
Supplement: Additional file 1 — SBRT Archive. An archive of the current version of the Systems Biology Research Tool. [file 1752-0509-2-55-S1.zip › sbrt-1.4.0/doc/developers_guide/api/sbrt/shell/mng/fba/NetworkInfoManager.html]

NetworkInfoManager


|  |  |  |
| --- | --- | --- |
| **Overview** \| **Package** \| **Class** \| **Use** \| **Tree** \| **Deprecated** \| **Index** \| **Help** | |  |
| **PREV CLASS**   **NEXT CLASS** | **FRAMES**    **NO FRAMES**     **All Classes** |  |
| SUMMARY: NESTED \| FIELD \| CONSTR \| METHOD | DETAIL: FIELD \| CONSTR \| METHOD |  |


---


## sbrt.shell.mng.fba Class NetworkInfoManager

```
java.lang.Object
  sbrt.shell.mng.AbstractProcessManager
      sbrt.shell.mng.fba.FbaManager
          sbrt.shell.mng.fba.NetworkInfoManager
```

**All Implemented Interfaces:**: KernelProcessManager<java.lang.String,java.lang.String>, ProcessManager<java.lang.String,java.lang.String>

---

``` public final class NetworkInfoManager extends FbaManager ```

This class is used to manage fluxome information experiments.

**Author:**
:   This class was written and documented by Jeremiah Wright while in the
    Wagner lab.

---

| **Field Summary** | |
| --- | --- |
| `static java.lang.String` | `CATALYST_FILE_NAME`             The keyword used to denote teh name of the file to which all catalysts will be written. |
| `static java.lang.String` | `CHEM_SPECIES_FILE_NAME`             The keyword used to denote the name of the file to which the chemical species of a fluxome will be written. |
| `static java.lang.String` | `EQUATION_FILE_NAME`             The keyword used to denote the name of the file to which the system of linear equations formed by a fluxome will be written. |
| `static java.lang.String` | `EQUIV_RXN_FILE_NAME`             The keyword used to denote the name of the file to which stoichiometrically equivalent reactions will be written. |
| `static java.lang.String` | `MATRIX_FILE_NAME`             The keyword used to denote the name of the file to which the stoichiometry matrix of a fluxome will be written. |
| `static UnmodifiableSet<java.lang.String>` | `optionalKeywords`             The set of keywords understood, but not required, by instances of this class. |
| `static java.lang.String` | `REMOVE_REDUNDANT_REACTIONS`             The keyword used to denote whether or not redundant reactions should be removed. |
| `static UnmodifiableSet<java.lang.String>` | `requiredKeywords`             The set of keywords required to be present in the `ManagerInput` objects supplied to instances of this class. |
| `static java.lang.String` | `RXN_NAME_FILE_NAME`             The keyword used to denote the name of the file to which the names of reactions of a fluxome will be written. |
| `static java.lang.String` | `RXN_OUTPUT_FILE_NAME`             The keyword used to denote the name of the file to which a fluxome will be written. |

**Fields inherited from class sbrt.shell.mng.fba.FbaManager**

`CONSTRAINT_TOLERANCE, DEFAULT_CONSTRAINT_TOLERANCE, FLUX_VECTOR_FILE, FLUX_VECTOR_FILE_NAME, RXN_CATALYST_FILE, RXN_FILE, SYSTEM_SOLUTION_FILE`

**Fields inherited from class sbrt.shell.mng.AbstractProcessManager**

`ALGORITHM, COMPLETED, DATA_HEADERS, DEV_NULL, ELAPSED_TIME, FILE_FORMAT, INPUT_FILE, INPUT_FILE_FORMAT, INPUT_FILE_NAME_FILE, ITERATIONS, OUTPUT_FILE_FORMAT, OUTPUT_FILE_NAME, OUTPUT_FILE_NAME_FILE, PERCENT, PERCENT_COMPLETED, SEED, ZERO_CUTOFF`


**Constructor Summary**

`NetworkInfoManager()`


| **Method Summary** | |
| --- | --- |
| `void` | `execute(java.io.PrintWriter out)`             Executes the process this process manager controls, and writes the status or results of this process to the provided print writer. |
| `UnmodifiableSet<java.lang.String>` | `getOptionalKeys()`             Returns the set of keywords the provided `ManagerInput` object does not necessarily have to contain. |
| `NetworkInfoProcess` | `getProcess()`             Returns the process this process manager controls. |
| `UnmodifiableSet<java.lang.String>` | `getRequiredKeys()`             Returns the set of keywords the provided `ManagerInput` object must contain. |
| `static void` | `printBasicInfo(Fluxome fluxome, java.io.PrintWriter out)`             Prints basic information about the provided fluxome. |
| `void` | `setInput(ManagerInput<java.lang.String,java.lang.String> input, java.io.PrintWriter out)`             Uses the provided input to prepare this process manager for execution. |

**Methods inherited from class sbrt.shell.mng.fba.FbaManager**

`getCatalysts, getCatalysts, getConstraintTolerance, getConstraintTolerance, getFluxome, getFluxome, getFluxome, getFluxome, getFluxome, getFluxomeSolution, getMultiFluxVectorOutputFile, getMultiFluxVectorOutputFile, getRxnNameExprFormat, getRxnNameHeaders, getRxnNameHeaders, getSingleFluxVectorOutputFile, getSingleFluxVectorOutputFile, printFluxomeInfo, writeFluxome, writeFluxome`

**Methods inherited from class sbrt.shell.mng.AbstractProcessManager**

`check, closeFile, closeRm, execute, getFileFormat, getFileFormat, getFileNameSource, getIterations, getSeed, getTextFileFormat, getTextFileFormat, getZeroCutoff, getZeroCutoff, parseInt, parseLong, printProgress, printProgress, printProgress, runProcess, setInput`

**Methods inherited from class java.lang.Object**

`clone, equals, finalize, getClass, hashCode, notify, notifyAll, toString, wait, wait, wait`

**Methods inherited from interface sbrt.shell.mng.ProcessManager**

`check, execute, setInput`

**Field Detail**

### REMOVE\_REDUNDANT\_REACTIONS

```
public static final java.lang.String REMOVE_REDUNDANT_REACTIONS
```

:   The keyword used to denote whether or not redundant reactions should be
    removed.

    **See Also:**: Constant Field Values

---


### EQUIV\_RXN\_FILE\_NAME

```
public static final java.lang.String EQUIV_RXN_FILE_NAME
```

:   The keyword used to denote the name of the file to which
    stoichiometrically equivalent reactions will be written.

    **See Also:**: Constant Field Values

---


### EQUATION\_FILE\_NAME

```
public static final java.lang.String EQUATION_FILE_NAME
```

:   The keyword used to denote the name of the file to which the system of
    linear equations formed by a fluxome will be written.

    **See Also:**: Constant Field Values

---


### MATRIX\_FILE\_NAME

```
public static final java.lang.String MATRIX_FILE_NAME
```

:   The keyword used to denote the name of the file to which the
    stoichiometry matrix of a fluxome will be written.

    **See Also:**: Constant Field Values

---


### CHEM\_SPECIES\_FILE\_NAME

```
public static final java.lang.String CHEM_SPECIES_FILE_NAME
```

:   The keyword used to denote the name of the file to which the chemical species
    of a fluxome will be written.

    **See Also:**: Constant Field Values

---


### RXN\_NAME\_FILE\_NAME

```
public static final java.lang.String RXN_NAME_FILE_NAME
```

:   The keyword used to denote the name of the file to which the names of
    reactions of a fluxome will be written.

    **See Also:**: Constant Field Values

---


### RXN\_OUTPUT\_FILE\_NAME

```
public static final java.lang.String RXN_OUTPUT_FILE_NAME
```

:   The keyword used to denote the name of the file to which a fluxome will
    be written.

    **See Also:**: Constant Field Values

---


### CATALYST\_FILE\_NAME

```
public static final java.lang.String CATALYST_FILE_NAME
```

:   The keyword used to denote teh name of the file to which all catalysts
    will be written.

    **See Also:**: Constant Field Values

---


### requiredKeywords

```
public static final UnmodifiableSet<java.lang.String> requiredKeywords
```

:   The set of keywords required to be present in the `ManagerInput`
    objects supplied to instances of this class.

---


### optionalKeywords

```
public static final UnmodifiableSet<java.lang.String> optionalKeywords
```

:   The set of keywords understood, but not required, by instances of this
    class.


**Constructor Detail**

### NetworkInfoManager

```
public NetworkInfoManager()
```


**Method Detail**

### printBasicInfo

```
public static void printBasicInfo(Fluxome fluxome,
                                  java.io.PrintWriter out)
```

:   Prints basic information about the provided fluxome.

    :   **Parameters:**: `fluxome` - the fluxome for which basic information will be printed.: `out` - the print writer to which basic information will be printed.

---


### getRequiredKeys

```
public UnmodifiableSet<java.lang.String> getRequiredKeys()
```

:   Returns the set of keywords the provided `ManagerInput` object must
    contain.

    :   **Returns:**: the set of keywords the provided `ManagerInput` object must contain.

---


### getOptionalKeys

```
public UnmodifiableSet<java.lang.String> getOptionalKeys()
```

:   Returns the set of keywords the provided `ManagerInput` object does
    not necessarily have to contain.

    :   **Returns:**: the set of keywords the provided `ManagerInput` object does not necessarily have to contain.

---


### getProcess

```
public NetworkInfoProcess getProcess()
```

:   Returns the process this process manager controls.

    :   **Returns:**: the process this process manager controls. **Throws:**: `java.lang.IllegalStateException` - if no input was provided to this process manager via `AbstractProcessManager.setInput(ManagerInput)` or `setInput(ManagerInput, PrintWriter)`

---


### setInput

```
public void setInput(ManagerInput<java.lang.String,java.lang.String> input,
                     java.io.PrintWriter out)
```

:   Uses the provided input to prepare this process manager for execution. As
    the input is processed, informative messages are printed to the provided
    print writer.

    :   **Parameters:**: `input` - all of the information required to prepare this process manager for execution.: `out` - the print writer to which all messages will be printed.

---


### execute

```
public void execute(java.io.PrintWriter out)
```

:   Executes the process this process manager controls, and writes the status
    or results of this process to the provided print writer.

    :   **Parameters:**: `out` - the print writer where status messages will be printed. **Throws:**: `java.lang.IllegalStateException` - if no input was provided to this process manager via `AbstractProcessManager.setInput(ManagerInput)` or `setInput(ManagerInput, PrintWriter)`


---


|  |  |  |
| --- | --- | --- |
| **Overview** \| **Package** \| **Class** \| **Use** \| **Tree** \| **Deprecated** \| **Index** \| **Help** | |  |
| **PREV CLASS**   **NEXT CLASS** | **FRAMES**    **NO FRAMES**     **All Classes** |  |
| SUMMARY: NESTED \| FIELD \| CONSTR \| METHOD | DETAIL: FIELD \| CONSTR \| METHOD |  |


---
